# Supplementary material for: HIVprotI: an integrated web based platform for prediction and design of HIV proteins inhibitors
Source: J Cheminform. 2018 Mar 9;10:12. doi: 10.1186/s13321-018-0266-y (PMC5845081; doi:10.1186/s13321-018-0266-y)
Supplement: Supplementary file 2 — Additional file 2. Source code of HIVProtI web server. [file 13321_2018_266_MOESM2_ESM.zip › HIVprotI_Source-code/hivprotI/weka-3-6-6/doc/weka/gui/treevisualizer/Node.html]

Node


---


|  |  |  |
| --- | --- | --- |
| **Overview** \| **Package** \| **Class** \| **Tree** \| **Deprecated** \| **Index** \| **Help** \| **Weka's home** | |  |
| **PREV CLASS**   **NEXT CLASS** | **FRAMES**    **NO FRAMES**     **All Classes** |  |
| SUMMARY: NESTED \| FIELD \| CONSTR \| METHOD | DETAIL: FIELD \| CONSTR \| METHOD |  |


---


## weka.gui.treevisualizer Class Node

```
java.lang.Object
  weka.gui.treevisualizer.Node
```

---

``` public class Node extends java.lang.Object ```

This class records all the data about a particular node for displaying.

**Version:**
:   $Revision: 7059 $

**Author:**
:   Malcolm Ware (mfw4@cs.waikato.ac.nz)

---

**Constructor Summary**

`Node(java.lang.String label,
java.lang.String refer,
int backstyle,
int shape,
java.awt.Color color,
java.lang.String d)`
  
          This will setup all the values of the node except for its top and center.


| **Method Summary** | |
| --- | --- |
| `void` | `addChild(Edge v)`             Set the value of children. |
| `void` | `adjustCenter(double v)`             Will increase or decrease the postion of center. |
| `double` | `getCenter()`             Get the value of center. |
| `Edge` | `getChild(int i)`             Get the Edge for the child number 'i'. |
| `java.awt.Color` | `getColor()`             Get the value of color. |
| `static int` | `getCount(Node r, int n)`             Recursively finds the number of visible nodes there are (this may accidentally count some of the invis nodes). |
| `boolean` | `getCVisible()`             Get If this node's childs are visible. |
| `static int` | `getGCount(Node r, int n)`             Recursively finds the number of visible groups of siblings there are. |
| `static int` | `getHeight(Node r, int l)`             Recursively finds the number of visible levels there are. |
| `Instances` | `getInstances()`             This will return the Instances object related to this node. |
| `java.lang.String` | `getLabel()`             Get the value of label. |
| `java.lang.String` | `getLine(int n)`             Returns the text String for the specfied line. |
| `Edge` | `getParent(int i)`             Get the parent edge. |
| `java.lang.String` | `getRefer()`             Get the value of refer. |
| `boolean` | `getRoot()`             Get the value of root. |
| `int` | `getShape()`             Get the value of shape. |
| `double` | `getTop()`             Get the value of top. |
| `static int` | `getTotalCount(Node r, int n)`             Recursively finds the total number of nodes there are. |
| `static int` | `getTotalGCount(Node r, int n)`             Recursively finds the total number of groups of siblings there are. |
| `static int` | `getTotalHeight(Node r, int l)`             Recursively finds the total number of levels there are. |
| `boolean` | `getVisible()`             Get the value of visible. |
| `void` | `setCenter(double v)`             Set the value of center. |
| `void` | `setColor(java.awt.Color v)`             Set the value of color. |
| `void` | `setCVisible(boolean v)`             Sets all the children of this node either to visible or invisible |
| `void` | `setParent(Edge v)`             Set the value of parent. |
| `void` | `setRefer(java.lang.String v)`             Set the value of refer. |
| `void` | `setRoot(boolean v)`             Set the value of root. |
| `void` | `setShape(int v)`             Set the value of shape. |
| `void` | `setTop(double v)`             Set the value of top. |
| `java.awt.Dimension` | `stringSize(java.awt.FontMetrics f)`             This will return the width and height of the rectangle that the text will fit into. |

**Methods inherited from class java.lang.Object**

`equals, getClass, hashCode, notify, notifyAll, toString, wait, wait, wait`

**Constructor Detail**

### Node

```
public Node(java.lang.String label,
            java.lang.String refer,
            int backstyle,
            int shape,
            java.awt.Color color,
            java.lang.String d)
```

:   This will setup all the values of the node except for its top and center.

    **Parameters:**: `label` - The text for the node.: `refer` - The ID string for this node.: `backstyle` - The backstyle of this node.: `shape` - The shape of this node.: `color` - The color of this node.


**Method Detail**

### getInstances

```
public Instances getInstances()
```

:   This will return the Instances object related to this node.
    If it has not been allocated then that will be done also.

    :   **Returns:**: The Instances object.

---


### getCVisible

```
public boolean getCVisible()
```

:   Get If this node's childs are visible.

    :   **Returns:**: True if the childs are visible.

---


### setCVisible

```
public void setCVisible(boolean v)
```

:   Sets all the children of this node either to visible or invisible

    :   **Parameters:**: `v` - True if the children are to be visible

---


### getRefer

```
public java.lang.String getRefer()
```

:   Get the value of refer.

    :   **Returns:**: Value of refer.

---


### setRefer

```
public void setRefer(java.lang.String v)
```

:   Set the value of refer.

    :   **Parameters:**: `v` - Value to assign to refer.

---


### getShape

```
public int getShape()
```

:   Get the value of shape.

    :   **Returns:**: Value of shape.

---


### setShape

```
public void setShape(int v)
```

:   Set the value of shape.

    :   **Parameters:**: `v` - Value to assign to shape.

---


### getColor

```
public java.awt.Color getColor()
```

:   Get the value of color.

    :   **Returns:**: Value of color.

---


### setColor

```
public void setColor(java.awt.Color v)
```

:   Set the value of color.

    :   **Parameters:**: `v` - Value to assign to color.

---


### getLabel

```
public java.lang.String getLabel()
```

:   Get the value of label.

    :   **Returns:**: Value of label.

---


### stringSize

```
public java.awt.Dimension stringSize(java.awt.FontMetrics f)
```

:   This will return the width and height of the rectangle that the text
    will fit into.

    :   **Parameters:**: `f` - The size info for the Font. **Returns:**: A Dimension containing the size of the text.

---


### getLine

```
public java.lang.String getLine(int n)
```

:   Returns the text String for the specfied line.

    :   **Parameters:**: `n` - The line wanted. **Returns:**: The String corresponding to that line.

---


### getCenter

```
public double getCenter()
```

:   Get the value of center.

    :   **Returns:**: Value of center.

---


### setCenter

```
public void setCenter(double v)
```

:   Set the value of center.

    :   **Parameters:**: `v` - Value to assign to center.

---


### adjustCenter

```
public void adjustCenter(double v)
```

:   Will increase or decrease the postion of center.

    :   **Parameters:**: `v` - The amount to increase or decrease center by.

---


### getTop

```
public double getTop()
```

:   Get the value of top.

    :   **Returns:**: Value of top.

---


### setTop

```
public void setTop(double v)
```

:   Set the value of top.

    :   **Parameters:**: `v` - Value to assign to top.

---


### getVisible

```
public boolean getVisible()
```

:   Get the value of visible.

    :   **Returns:**: Value of visible.

---


### getRoot

```
public boolean getRoot()
```

:   Get the value of root.

    :   **Returns:**: True if has no parents.

---


### setRoot

```
public void setRoot(boolean v)
```

:   Set the value of root.

    :   **Parameters:**: `v` - Value to assign to root.

---


### getParent

```
public Edge getParent(int i)
```

:   Get the parent edge.

    :   **Parameters:**: `i` - The parent number to get. **Returns:**: The parent edge or NULL if it doesn't exist.

---


### setParent

```
public void setParent(Edge v)
```

:   Set the value of parent.

    :   **Parameters:**: `v` - Value to assign to parent.

---


### getChild

```
public Edge getChild(int i)
```

:   Get the Edge for the child number 'i'.

    :   **Parameters:**: `i` - The child number to get. **Returns:**: The child Edge or NULL if it doesn't exist.

---


### addChild

```
public void addChild(Edge v)
```

:   Set the value of children.

    :   **Parameters:**: `v` - Value to assign to children.

---


### getGCount

```
public static int getGCount(Node r,
                            int n)
```

:   Recursively finds the number of visible groups of siblings there are.

    :   **Parameters:**: `r` - The current Node upto.: `n` - The current number of groups there are. **Returns:**: The number of groups found so far.

---


### getTotalGCount

```
public static int getTotalGCount(Node r,
                                 int n)
```

:   Recursively finds the total number of groups of siblings there are.

    :   **Parameters:**: `r` - The current Node upto.: `n` - The current number of groups there are. **Returns:**: The number of groups found so far.

---


### getCount

```
public static int getCount(Node r,
                           int n)
```

:   Recursively finds the number of visible nodes there are (this may
    accidentally count some of the invis nodes).

    :   **Parameters:**: `r` - The current Node upto.: `n` - The current number nodes there are. **Returns:**: The number of nodes found so far.

---


### getTotalCount

```
public static int getTotalCount(Node r,
                                int n)
```

:   Recursively finds the total number of nodes there are.

    :   **Parameters:**: `r` - The current Node upto.: `n` - The current number nodes there are. **Returns:**: The number of nodes found so far.

---


### getHeight

```
public static int getHeight(Node r,
                            int l)
```

:   Recursively finds the number of visible levels there are.

    :   **Parameters:**: `r` - The current Node upto.: `l` - The curent level. **Returns:**: The max number of levels found so far.

---


### getTotalHeight

```
public static int getTotalHeight(Node r,
                                 int l)
```

:   Recursively finds the total number of levels there are.

    :   **Parameters:**: `r` - The current Node upto.: `l` - The curent level. **Returns:**: The max number of levels found so far.


---


|  |  |  |
| --- | --- | --- |
| **Overview** \| **Package** \| **Class** \| **Tree** \| **Deprecated** \| **Index** \| **Help** \| **Weka's home** | |  |
| **PREV CLASS**   **NEXT CLASS** | **FRAMES**    **NO FRAMES**     **All Classes** |  |
| SUMMARY: NESTED \| FIELD \| CONSTR \| METHOD | DETAIL: FIELD \| CONSTR \| METHOD |  |


---
